# Experiences of Psychotherapists With Remote Psychotherapy During the COVID-19 Pandemic: Cross-sectional Web-Based Survey Study

**DOI:** 10.2196/20246

**Published:** 2020-11-27

**Authors:** Elke Humer, Peter Stippl, Christoph Pieh, Rüdiger Pryss, Thomas Probst

**Affiliations:** 1 Department for Psychotherapy and Biopsychosocial Health Danube University Krems Krems Austria; 2 Austrian Federal Association for Psychotherapy Vienna Austria; 3 Institute of Clinical Epidemiology and Biometry University of Würzburg Würzburg Germany

**Keywords:** psychotherapists, remote psychotherapy, telephone, internet, experiences, expectations, COVID-19, telehealth, therapy, psychology

## Abstract

**Background:**

The current situation around the COVID-19 pandemic and the measures necessary to fight it are creating challenges for psychotherapists, who usually treat patients face-to-face with personal contact. The pandemic is accelerating the use of remote psychotherapy (ie, psychotherapy provided via telephone or the internet). However, some psychotherapists have expressed reservations regarding remote psychotherapy. As psychotherapists are the individuals who determine the frequency of use of remote psychotherapy, the potential of enabling mental health care during the COVID-19 pandemic in line with the protective measures to fight COVID-19 can be realized only if psychotherapists are willing to use remote psychotherapy.

**Objective:**

This study aimed to investigate the experiences of psychotherapists with remote psychotherapy in the first weeks of the COVID-19 lockdown in Austria (between March 24 and April 1, 2020).

**Methods:**

Austrian psychotherapists were invited to take part in a web-based survey. The therapeutic orientations of the psychotherapists (behavioral, humanistic, psychodynamic, or systemic), their rating of the comparability of remote psychotherapy (web- or telephone-based) with face-to-face psychotherapy involving personal contact, and potential discrepancies between their actual experiences and previous expectations with remote psychotherapy were assessed. Data from 1162 psychotherapists practicing before and during the COVID-19 lockdown were analyzed.

**Results:**

Psychotherapy conducted via telephone or the internet was reported to not be totally comparable to psychotherapy with personal contact (*P*<.001). Psychodynamic (*P*=.001) and humanistic (*P*=.005) therapists reported a higher comparability of telephone-based psychotherapy to in-person psychotherapy than behavioral therapists. Experiences with remote therapy (both web- and telephone-based) were more positive than previously expected (*P*<.001). Psychodynamic therapists reported more positive experiences with telephone-based psychotherapy than expected compared to behavioral (*P*=.03) and systemic (*P*=.002) therapists. In general, web-based psychotherapy was rated more positively (regarding comparability to psychotherapy with personal contact and experiences vs expectations) than telephone-based psychotherapy (*P*<.001); however, psychodynamic therapists reported their previous expectations to be equal to their actual experiences for both telephone- and web-based psychotherapy.

**Conclusions:**

Psychotherapists found their experiences with remote psychotherapy (ie, web- or telephone-based psychotherapy) to be better than expected but found that this mode was not totally comparable to face-to-face psychotherapy with personal contact. Especially, behavioral therapists were found to rate telephone-based psychotherapy less favorably than therapists with other theoretical backgrounds.

## Introduction

### Background

Remote psychotherapy, in which psychotherapy is provided from a distance, includes a broad range of technologies, encompassing the use of telephones, videoconferencing, and email [1]. Mental health care conducted remotely (ie, via videoconferencing) has rapidly evolved worldwide as a technology, as it enables the direct delivery of real-time psychotherapy to patients [2]. The benefits of remote psychotherapy derive from the improved access to psychotherapy by providing mental health care services to patients who face logistical and stigma-related barriers to receiving face-to-face treatment [3].

The current situation around the COVID-19 pandemic and the measures necessary to fight it have further accelerated the rapid expansion of the technology of remote psychotherapy [4]. This is mainly because the traditional form of face-to-face psychotherapy conducted in person contrasts with the efforts to contain the COVID-19 pandemic, including social distancing, isolation, and quarantine [5]. However, the observed increase in mental health problems during the COVID-19 outbreak additionally enhances the general need for mental health care during and after the COVID-19 pandemic [6-8]. A recent review reported that the mental health problems related to quarantine include a high prevalence of psychological distress, depression, anxiety, and trauma-related disorders [6]. Thus, this public health emergency is enhancing the necessity to provide mental health care while adhering to efforts to contain the COVID-19 pandemic [9-11].

Due to the required reduction of personal contacts, psychotherapists are confronted with major challenges to delivery of care. In general, psychotherapists are the individuals who determine the frequency of use of remote psychotherapy. Thus, the potential of increasing access to mental health care during the COVID-19 pandemic while adhering to the protective measures to fight COVID-19 can be realized only if psychotherapists are willing to use remote psychotherapy. In this regard, it is important to investigate whether psychotherapists evaluate remote psychotherapy to be equal to face-to-face psychotherapy. Moreover, the potential of psychotherapists who have experience with remote psychotherapy to revise their attitudes based upon their recent experiences should be explored. A previous study suggested that health care providers who used telemedicine methods for the first time had more positive attitudes afterward [12]. Adequate mental health care is of high importance during the COVID-19 pandemic, and the provision of psychotherapy at a safe distance seems to be the obvious solution to ensure sufficient psychotherapeutic support. Thus, exploring the attitudes and experiences of psychotherapists toward remote psychotherapy is essential to improve the accessibility of mental health care systems during and after COVID-19.

### Prior Work

In general, videoconferencing offers great potential for delivering psychotherapy from distance during the COVID-19 pandemic, as some evidence indicates comparable outcomes of providing psychotherapy remotely via the internet to in-person psychotherapy [13-15]. However, older technologies such as telephonic communication also offer immediate and easy-to-use ways to provide mental health care remotely. Moreover, equal effectiveness of telephone-based psychotherapy compared to face-to-face psychotherapy has been observed [16]. Nevertheless, despite research indicating comparable outcomes of providing psychotherapy remotely via telephone or the internet to providing in-person psychotherapy, psychotherapists have expressed some reservations about remote psychotherapy [17]. In general, psychotherapists seem to be more skeptical regarding remote psychotherapy than patients [2], who also show higher satisfaction with remote psychotherapy than therapists [18,19]. For example, while therapists rated the therapeutic relationship lower for videoconferencing than for in-person sessions, from the patients’ perspective, no differences between these modalities were observed [20]. Moreover, technical difficulties seem to be experienced as more problematic by therapists than by patients [21].

Overall, several previous studies focused on the attitudes, experiences, and effectiveness of remote psychotherapy at the patient level; however, less is known about the therapists’ perspective. In general, most studies comparing therapists’ attitudes toward remote psychotherapy and face-to-face therapy found a preference for conducting therapy in person [2].

The acceptance and use of remote technologies by therapists may be affected by their theoretical background. Although there are hundreds of psychotherapeutic approaches and different ways to categorize them, they can be broadly categorized into four general schools of thought: behavioral, humanistic, psychodynamic, and systemic approaches. In brief, behavioral approaches mainly rely on behavioral techniques to change maladaptive patterns of behavior or thoughts to improve emotional responses and behaviors [22,23]. Humanistic or “experiential” psychotherapies are based on humanistic psychology, focusing mainly on human development and individual needs, with an emphasis on positive growth and subjective meaning [24]. Psychodynamic approaches focus on revealing or interpreting unconscious conflicts, which are thought to cause mental disorders [25]. In contrast, systemic therapy focuses on the interactions of groups such as families, as well as their dynamics and patterns, rather than addressing people individually. Systemic therapy seeks to identify and address stagnant patterns of behavior in groups [26,27]. Contrasting results have been obtained regarding the moderating role of these therapeutic orientations. Previous studies reported that psychodynamic orientation was related to more negative attitudes toward psychotherapy provided remotely [28-30], while behavioral orientations were found to be related to a more positive attitude toward remote psychotherapy [29,30]. Some research also indicates a higher acceptance of telehealth in therapists with systemic orientations compared to psychodynamic or existential orientations [29]. However, other studies observed no relationship between therapeutic orientation and attitudes toward remote psychotherapy [31,32].

To the best of our knowledge, there are no previous studies on whether and to what degree psychotherapists perceive discrepancies in their actual experiences and previous expectations with remote psychotherapy in situations requiring a rapid adaption of therapeutic settings due to an ongoing public health emergency such as the COVID-19 outbreak. Therefore, the current study aimed to investigate how comparable psychotherapists experience remote psychotherapy compared to face-to-face psychotherapy and whether their actual experiences differ from their expectations. Furthermore, we were interested in potential differences among therapeutic orientations.

The COVID-19 lockdown in Austria became obligatory on March 16, 2020 [33-35]. In general, entering public places was strictly prohibited. People were only permitted to leave their homes if they had a good reason for doing so. At the time of the study, the only exceptions to the ban on entering public places were to avert immediate danger to life, limb, or property; to fulfill work responsibilities, although wherever possible, people should work from home; to meet necessary basic needs of daily life (eg, grocery shopping, visiting pharmacies, withdrawing money from cash machines, physician visits, medical treatments or therapy, pet maintenance); to take care of or support vulnerable people; and to practice low-risk sports (eg, walking or jogging), but only alone, with other people from one’s own household, or with pets. For these exceptions, it was necessary to maintain a minimum safe distance of 1 m between people. Certain areas in Austria were under quarantine at the time of the study and had even stronger restrictions.

At the time of the study, an official Austrian guideline addressing the conduction of psychotherapy via the internet rejected this treatment modality [36]. However, health insurance started to cover the costs for telephone- and web-based psychotherapy during the COVID-19 pandemic in Austria.

### Hypotheses

Based on the aforementioned literature, the following research questions (RQs) and hypotheses were addressed in the present study.

RQ 1: How do psychotherapists in the early weeks of the COVID-19 lockdown rate the comparability of telephone-based therapy to in-person psychotherapy?

RQ 1a: Do therapists rate telephone-based psychotherapy comparably to in-person psychotherapy? This RQ tested the hypothesis that psychotherapists would not rate telephone-based therapy to be equal to in-person therapy.

RQ 1b: Does the therapeutic orientation of the therapist affect the rating? We hypothesized that behavioral therapists would rate the comparability of telephone-based psychotherapy more positively compared to other therapeutic orientations. Moreover, we hypothesized that psychodynamic therapists would rate the comparability lower than other therapeutic orientations.

RQ 2: How much more negatively or positively do psychotherapists rate their actual experiences with telephone-based therapy in the early weeks of the COVID-19 lockdown compared to their previous expectations?

RQ 2a: Do the actual experiences of psychotherapists regarding telephone-based psychotherapy differ from their previous expectations? This RQ tested the hypothesis that therapists would rate their actual experiences with telephone-based psychotherapy higher than they previously expected.

RQ 2b: Is the discrepancy between actual experiences and previous expectations concerning telephone-based psychotherapy different between therapeutic orientations? We hypothesized that behavioral therapists would show a smaller discrepancy between their actual experiences and their previous expectations, whereas psychodynamic therapists would show the largest discrepancy.

RQ 3: How do psychotherapists in the early weeks of the COVID-19 lockdown rate the comparability of web-based therapy to in-person psychotherapy?

RQ 3a: Do therapists rate web-based psychotherapy comparably to in-person psychotherapy? This RQ tested the hypothesis that psychotherapists would not rate web-based therapy to be equal to in-person therapy.

RQ 3b: Does the therapeutic orientation of the psychotherapist affect the rating? We hypothesized that behavioral therapists would rate the comparability of web-based interventions more positively than therapists with other therapeutic orientations. Moreover, we hypothesized that psychodynamic therapists would rate the comparability lowest compared to the other therapeutic orientations.

RQ 4: How much more negatively or positively do psychotherapists rate their actual experiences with web-based therapy in the early weeks of the COVID-19 lockdown compared to their previous expectations?

RQ 4a: Do the actual experiences of psychotherapists regarding web-based psychotherapy differ from their previous expectations? This RQ tested the hypothesis that psychotherapists would rate their actual experiences with web-based therapy more positively compared to their previous expectations.

RQ 4b: Is the discrepancy between actual experiences and previous expectations different between therapeutic orientations? We hypothesized that behavioral therapists would show a smaller discrepancy between their actual experiences and their previous expectations, whereas psychodynamic therapists would show the largest discrepancy.

RQ 5: Does the format of remote psychotherapy (telephone or internet) affect the rating of psychotherapists regarding the comparability between remote psychotherapy and in-person psychotherapy? Moreover, is there an interaction between the comparability of telephone-based psychotherapy versus the comparability of web-based psychotherapy with the therapists’ therapeutic orientations? We had no specific hypothesis here.

RQ 6: Does the discrepancy between actual experiences and previous expectations regarding telephone-based psychotherapy differ from the discrepancy between actual experiences and previous expectations regarding web-based psychotherapy? In addition, is there an interaction between actual experiences compared to previous expectations for telephone-based psychotherapy versus actual experiences compared to previous expectations for web-based psychotherapy and therapeutic orientation? We had no specific hypothesis here.

## Methods

### Study Design

To investigate the use of remote psychotherapy during the first weeks of the COVID-19 lockdown in Austria, we conducted a cross-sectional survey from March 24 to April 1, 2020. The survey was designed in the Research Electronic Data Capture (REDCap) application and comprised 79 items focusing on the changes in the provision of psychotherapy, experiences with remote psychotherapy, fear of COVID-19 infection, adherence to protective measures against COVID-19 infection, perceived stress, job-related anxiety, and resilience, among others. More details on the conduction of the web-based survey have been published recently [37,38].

### Study Population

Eligible participants included all licensed Austrian psychotherapists registered in the list of psychotherapists of the Austrian Federal Ministry of Social Affairs, Health, Care, and Consumer Protection. All psychotherapists registered in the list who provided a valid email address (approximately 6000 out of 9319 licensed psychotherapists in total) were invited to take part in the survey. A web-based invitation with a link to the web-based survey was sent to the therapists by the last author in cooperation with the Austrian Federal Association for Psychotherapy.

### Measures

The following items were analyzed in the current study: demographic information (gender, age) as well as items regarding educational and professional background. More specifically, the psychotherapists were asked about their years in the profession (ie, years since becoming accredited as a psychotherapist) and about their psychotherapy method (in Austria, there are 23 accredited therapeutic methods [39]); the latter was categorized in one of the four eligible therapeutic orientations in Austria (behavioral, humanistic, psychodynamic, and systemic) for the present study.

Four items focused on the expectations of the psychotherapists regarding telephone- and web-based therapies. These questions were only asked to psychotherapists who were treating at least one patient before the COVID-19 lockdown as well as after the COVID-19 lockdown.

Psychotherapists were asked to rate whether they could treat their patients in a comparable manner with remote therapy compared to in-person therapy on a sliding scale ranging from 0 (not comparable at all) to 100 (totally comparable). Two separate questions were asked, one on telephone-based psychotherapy, and the other on web-based psychotherapy. The questions on the comparability of telephone- or web-based psychotherapy were only asked of psychotherapists who treated at least one patient via telephone or via the internet either before the COVID-19 lockdown or during the COVID-19 lockdown.

Psychotherapists were asked to rate whether their actual experiences with remote psychotherapy during the actual situation around COVID-19 matched their previous expectations regarding remote psychotherapy on a sliding scale ranging from 0 (significantly more negative than expected) to 100 (significantly more positive than expected). Again, two separate questions were asked, focusing either on telephone- or web-based psychotherapy. The questions regarding actual experiences versus previous expectations for telephone- or web-based psychotherapy were only asked of psychotherapists who started to treat patients via telephone or the internet during the COVID-19 lockdown (ie, no patients before the COVID-19 lockdown via telephone or internet and at least one patient during the COVID-19 via telephone or the internet). Only “new starters” were asked these questions because they were the only respondents to show a change of attitude in a previous study focusing on health professionals in general [12].

### Statistical Analysis

Statistical analyses were performed with SPSS version 25 (IBM Corporation).

Descriptive statistics were calculated to characterize the participants. To evaluate differences in sociodemographic characteristics, univariate analysis of variance (ANOVA) and chi-square tests were conducted. Bonferroni corrections were applied for the pairwise post-hoc tests.

The comparison of whether telephone- or web-based psychotherapy was regarded to be equal to psychotherapy in personal contact (RQ 1a, RQ 3a) was conducted using *t* tests; the respective ratings were compared with a value of 100, as a value of 100 represents the maximum score of the used sliding scale and means “totally comparable to psychotherapy in personal contact.”

To evaluate possible discrepancies between actual experiences and previous expectations regarding the use of the telephone or internet for psychotherapy (RQ 2a, RQ 4a), the ratings were compared against a value of 50, as a value of 50 indicates “previous expectations=actual experiences.”

To address RQ 1b, 2b, 3b, and 4b, univariate ANOVA was performed to investigate potential differences among the four therapeutic orientations of the psychotherapists (behavioral, humanistic, psychodynamic, and systemic) in the dependent variables (comparability of telephone- or web-based therapy to face-to-face therapy, actual experiences vs previous expectations for telephone- or web-based therapy). Bonferroni corrections were used for post-hoc comparisons.

To address RQ 5 and RQ 6, mixed ANOVA was performed with one within-subject variable (two levels of treatment format: telephone-based psychotherapy and web-based psychotherapy) and one between-subject variable (four levels of psychotherapeutic orientation: psychodynamic, humanistic, systematic, behavioral). For variables with a significant interaction between treatment format and therapeutic orientation, Bonferroni corrections were applied for the pairwise post-hoc tests. The dependent variables were the comparability of telephone- or web-based therapy to face-to-face therapy and actual experiences versus previous expectations for telephone- or web-based therapy.

To evaluate whether the results are robust when accounting for the found differences in age and professional experience between the therapeutic orientations, we used analysis of covariance (ANCOVA) tests.

All statistical tests were two-tailed, with a *P* value <.05 indicating statistical significance.

## Results

### Participant Characteristics

In total, 1547 psychotherapists participated in the survey, corresponding to a response rate of approximately 25%. For the present study, only therapists practicing as psychotherapists in the months before the COVID-19 pandemic as well during the COVID-19 lockdown were analyzed to investigate the RQs. The description of the sample and comparisons between the included and excluded participants are shown in Table 1. There was a significant difference in age (*P*=.02); the excluded psychotherapists were older than the included psychotherapists. However, the effect size of this difference was small (*g*=0.14).

**Table 1:** Sociodemographic characteristics of the surveyed psychotherapists included in or excluded from the investigation (N=1547). Only respondents who practiced as psychotherapists before and during the COVID-19 pandemic were included.

Variable		Included participants (n=1162)	Excluded participants (n=385)	Statistical evaluation	*P* value
**Gender**				χ^2^_1_=2.454	.12
	Female, n (%)	891 (76.7)	280 (72.7)		
	Male, n (%)	271 (23.3)	105 (27.3)		
Age (years), mean (SD)		51.3 (9.67)	52.7 (9.69)	t_1545_=2.409	.02
**Therapeutic orientation^a^**				χ^2^_3_=3.662	.30
	Psychodynamic, n (%)	234 (20.3)	90 (23.7)		
	Humanistic, n (%)	546 (47.4)	170 (44.9)		
	Systemic, n (%)	252 (21.9)	88 (23.2)		
	Behavioral, n (%)	120 (10.4)	31 (8.2)		
Professional experience (years),^b^ mean (SD)		11.2 (9.21)	11.1 (9.19)	t_1517_=–0.259	.80

^a^Data were available for 1152 of the included and 379 of the excluded participants.

^b^Data were available for 1143 of the included and 376 of the excluded participants.

For the 1162 included psychotherapists, we also investigated the differences between the therapeutic orientations in terms of gender, age, and professional experience (Table 2), with the following results:

Behavioral therapists were significantly younger than humanistic, systemic, and psychodynamic therapists (*P*≤.001 for all pairwise post-hoc comparisons). Similarly, behavioral therapists had significantly fewer years in the profession than humanistic, systemic, and psychodynamic therapists (*P*≤.04 for all pairwise post-hoc comparisons).

There was no difference regarding gender among the therapeutic orientations (*P=*.42).

**Table 2:** Sociodemographic characteristics of the psychotherapists included in the study in relation to their therapeutic orientation (n=1162).

Variable		n (%)	Mean years (SD)	Statistical evaluation	*P* value
**Female gender**				χ^2^_3_=2.797	.42
	Behavioral	95 (79.2)	N/A^a^		
	Humanistic	409 (74.9)	N/A		
	Psychodynamic	187 (79.9)	N/A		
	Systemic	195 (77.4)	N/A		
**Age**				*F*_3,1148_=11.140	*<*.001
	Behavioral	120 (10.4)	46.8 (9.82)		
	Humanistic	546 (47.4)	51.7 (9.35)		
	Psychodynamic	234 (20.3)	52.7 (9.96)		
	Systemic	252 (21.9)	50.9 (9.37)		
**Professional experience**				*F*_3,1129_=4.951	.002
	Behavioral	117 (10.3)	8.4 (8.17)		
	Humanistic	536 (47.3)	11.1 (9.23)		
	Psychodynamic	229 (20.2)	12.4 (9.61)		
	Systemic	251 (22.2)	11.2 (8.62)		

^a^N/A: not applicable.

### Results for RQ 1

RQ 1a: Psychotherapists who treated at least one patient with telephone-based psychotherapy either before or during the COVID-19 lockdown (n=1015) stated that telephone-based psychotherapy is not totally comparable to in-person psychotherapy. The mean score of 50.2 (SD 26.00) was significantly lower than 100, *T*_1014_=–61.07, *P*<.001.

RQ 1b: The therapeutic orientation affected the rating of whether telephone-based psychotherapy is comparable to face-to-face psychotherapy (*P=*.001). As summarized in Table 3, the highest values were observed for psychodynamic therapists, while the lowest values were observed for behavioral therapists. Post-hoc tests revealed a significantly higher rating by psychodynamic therapists compared to behavioral therapists (*P*=.001) and a higher rating by humanistic therapists compared to behavioral therapists (*P*=.005). No differences were observed for the systemic orientation compared to the other three orientations.

**Table 3:** Psychotherapists’ ratings of their ability to treat patients via telephone comparable to in-person therapy on a sliding scale ranging from 0 (not comparable at all) to 100 (totally comparable) (n=1007; *F*_3,1003_=5.626, *P*=.001).

Therapeutic orientation	n (%)	Mean (SD)
Behavioral	101 (10.0)	41.7 (24.97)
Humanistic	468 (46.5)	51.2 (25.64)
Psychodynamic	205 (20.4)	53.9 (25.78)
Systemic	233 (23.1)	48.6 (26.57)

### Results for RQ 2

#### RQ 2a

Psychotherapists who started to use telephone for psychotherapy during the COVID-19 lockdown (n=782) stated that their actual experiences with telephone-based psychotherapy were better than previously expected. The mean score of 64.2 (SD 19.12) was significantly higher than 50 (t_781_=20.707, *P*<.001).

#### RQ 2b

The therapeutic orientation affected the rating of whether telephone-based psychotherapy was experienced differently than expected (*P=*.001). As summarized in Table 4, the highest values were observed for psychodynamic therapists, while the lowest values were observed for behavioral therapists. Post-hoc tests revealed a significantly higher rating in psychodynamic compared to behavioral therapists, *P*=.03, and systemic therapists, *P*=.002. No differences were observed for the humanistic orientation compared to the other three orientations.

**Table 4:** Psychotherapists’ actual experiences with telephone-based psychotherapy in relation to their previous expectations rated on a sliding scale ranging from 0 (significantly more negative than expected) to 100 (significantly more positive than expected) (n=777; *F*_3,773_=5.243, *P*=.001).

Therapeutic orientation	n (%)	Mean (SD)
Behavioral	73 (9.4)	60.6 (21.87)
Humanistic	370 (47.6)	64.8 (17.48)
Psychodynamic	157 (20.2)	68.3 (18.98)
Systemic	177 (22.8)	60.8 (20.64)

### Results for RQ 3

#### RQ 3a

Psychotherapists who treated at least one patient with web-based psychotherapy either before or during the COVID-19 lockdown (n=733) stated that web-based psychotherapy is not comparable to in-person psychotherapy. The mean score of 61.5 (SD 24.38) was significantly lower than 100 (t_732_=–42.779, *P*<.001).

#### RQ 3b

The rating of whether web-based psychotherapy is comparable to face-to-face psychotherapy did not differ between therapeutic orientations (*P=*.88), as summarized in Table 5.

**Table 5:** Psychotherapists’ rating of their ability to treat patients via the internet comparable to in-person therapy on a sliding scale ranging from 0 (not comparable at all) to 100 (totally comparable) (n=728; *F*_3,724_=0.226, *P*=.88).

Therapeutic orientation	n (%)	Mean (SD)
Behavioral	85 (11.7)	60.1 (23.90)
Humanistic	352 (48.4)	61.6 (24.35)
Psychodynamic	128 (17.6)	61.3 (24.14)
Systemic	163 (22.4)	62.7 (24.74)

### Results for RQ 4

#### RQ 4a

Psychotherapists who started to use internet for psychotherapy during the COVID-19 lockdown (n=614) stated that their actual experiences with web-based psychotherapy were better than previously expected. The mean score of 69.0 (SD 18.87) was significantly higher than 50 (t_613_=24.88, *P*<.001).

#### RQ 4b

The discrepancy between the actual experiences and previous expectations regarding web-based psychotherapy did not differ concerning the therapeutic orientation of the psychotherapists (*P=*.71). The mean scores (SD) are summarized in Table 6.

**Table 6:** Psychotherapists’ experiences with web-based psychotherapy in relation to their previous expectations on a sliding scale ranging from 0 (significantly more negative than expected) to 100 (significantly more positive than expected) (n=610; *F*_3,606_=0.459, *P*=.71).

Therapeutic orientation	n (%)	Mean (SD)
Behavioral	72 (11.8)	67.7 (23.18)
Humanistic	303 (49.7)	68.7 (17.75)
Psychodynamic	100 (16.4)	68.7 (18.13)
Systemic	135 (22.1)	70.6 (19.39)

### Results for RQ 5

Psychotherapists of the four orientations who treated at least one patient via telephone and at least one patient via the internet either before or during the COVID-19 lockdown (n=611) rated web-based psychotherapy to be more comparable to face-to-face psychotherapy than telephone-based psychotherapy (*F*_1,60_*_7_*=144.214, *P<*.001). No interaction between psychotherapy format (internet or telephone) and therapeutic orientation was observed (*F*_3,60_*_7_*=1.729, *P=*.16; Table 7).

**Table 7:** Psychotherapists’ ratings of their ability to comparably treat their patients via telephone or the internet, respectively, compared to in-person therapy on a sliding scale ranging from 0 (not comparable at all) to 100 (totally comparable) (n=611; *F*_3,607_=1.729, *P*=.16).

Therapeutic orientation	Telephone-based therapy		Web-based therapy	
	n (%)	Mean (SD)	n (%)	Mean (SD)
Behavioral	69 (11.3)	45.0 (24.25)	69 (11.3)	59.2 (24.51)
Humanistic	289 (47.3)	51.6 (24.41)	289 (47.3)	63.0 (23.41)
Psychodynamic	105 (17.2)	52.6 (24.74)	105 (17.2)	60.3 (24.36)
Systemic	148 (24.2)	50.1 (25.49)	148 (24.2)	62.8 (23.92)

### Results for RQ 6

Psychotherapists of the four therapeutic orientations who started to treat at least one patient via telephone and at least one patient via the internet during the COVID-19 lockdown (n=422) reported that their actual experiences with web-based psychotherapy were more positive than their actual experiences with telephone-based psychotherapy compared to their previous expectations (*F*_1,418_=22.680; *P<*.001). An interaction between the psychotherapy format (internet or telephone) and the therapeutic orientation was observed (*F*_3,418_=4.862, *P=*.002; Table 8). Post-hoc comparisons revealed that behavioral (*P*=.03), humanistic (*P*=.005), and systemic (*P*<.001) psychotherapists reported that their actual experiences compared to their previous expectations were more positive for web-based therapy than for telephone-based therapy. However, the discrepancy between actual experiences and previous expectations with remote psychotherapy was not different between web- and telephone-based psychotherapy among psychodynamic therapists (*P*>.99).

**Table 8:** Psychotherapists’ experiences with telephone- and web-based psychotherapy, respectively, compared to previous expectations rated on a sliding scale ranging from 0 (significantly more negative than expected) to 100 (significantly more positive than expected) (n=422; *F*_3,418_=4.862, *P*=.002).

Therapeutic orientation	Telephone-based therapy		Web-based therapy	
	n (%)	Mean (SD)	n (%)	Mean (SD)
Behavioral	45 (10.7)	63.2 (22.21)	45 (10.7)	69.0 (22.05)
Humanistic	202 (47.9)	66.1 (16.91)	202 (47.9)	69.7 (17.58)
Psychodynamic	72 (17.1)	68.3 (16.58)	72 (17.1)	68.3 (18.22)
Systemic	103 (24.4)	61.7 (21.13)	103 (24.4)	71.6 (19.95)

## Discussion

### Principal Results

This survey explored the experiences of psychotherapists in Austria with remote psychotherapy during the COVID-19 lockdown. Remote psychotherapy was reported to not be totally comparable to in-person psychotherapy, although the psychotherapists reported that their actual experiences with remote psychotherapy were better than expected. Psychodynamic and humanistic therapists rated telephone-based psychotherapy more comparably to face-to-face psychotherapy than behavioral therapists. The actual experiences with telephone-based psychotherapy differed more positively from previous expectations among psychodynamic therapists than among behavioral and systemic therapists. Web-based psychotherapy was rated to be more comparable to in-person psychotherapy than telephone-based psychotherapy, and this did not differ between therapeutic orientations. Also, the actual experiences of most psychotherapists were more positive than their previous expectations for web-based psychotherapy than for telephone-based psychotherapy; however, this was not the case for psychodynamic therapists.

### Limitations

There are several limitations in this study. One limitation is that the survey was conducted on the web; this may have caused some respondent bias, such as higher participation of psychotherapists with a higher preference for new technologies. This bias may have contributed to the finding that providing psychotherapy remotely was rated more positively than expected. The web-based conduction of the survey may also have caused a selection bias toward participation by fewer older psychotherapists [40]. A further limitation is that “telephone-based” and “web-based” are rather broad categories for treatment formats, and more detailed information about these formats, such as use of videoconferencing, chats, apps, or email, was not assessed. Another shortcoming is that the comparability of remote psychotherapy to in-person psychotherapy was operationalized by a survey conducted among psychotherapists, while no patient rating surveys or effectiveness measures were conducted. For future studies, the patients’ perspective, as well as outcome measures, should also be evaluated. These studies are not easily performed with web-based surveys; randomized controlled trials to evaluate efficacy need considerable planning time and thus were not feasible to evaluate remote psychotherapy during the first weeks of the COVID-19 lockdown. A further shortcoming is that the web-based conduction of the study prevented any measures of treatment adherence. Thus, it is not possible to say whether the therapeutic methods applied truly resembled the theoretical methods the therapists were trained to use. Moreover, the included participants are not representative of the excluded survey participants in age, as the excluded participants were approximately 1.5 years older (the effect size of this difference was rather small, with *g*=0.14). As the study was conducted in Austria, the results may only apply to countries with similar mental health care systems and similar therapeutic orientations among psychotherapists. In Austria, internet-based psychotherapy was rejected by an official guideline at the time of the study [34]. However, health insurance companies started to cover the costs of telephone- and web-based psychotherapy during the COVID-19 pandemic. Thus, the results are not directly comparable to countries that had already implemented mental eHealth solutions in routine psychotherapeutic practice.

### Comparison With Prior Work

Our results confirm the hypothesis that psychotherapy via telephone or the internet is not regarded to be completely comparable to in-person psychotherapy by therapists. In agreement, most studies investigating experience with remote psychotherapy reported that a minority of therapists experienced remote psychotherapy to be equal to face-to-face encounters [20,21,41]. Doubts regarding the comparability of remote psychotherapy with face-to-face psychotherapy arise from low performance expectancy, the lack of nonverbal communication, and difficulties in dealing with crises from a distance [17]. Also, the unsuitability of remote psychotherapy for all patients was identified as a disadvantage, and some therapists were concerned that remote therapy would be time-consuming or hinder the establishment of a therapeutic relationship [42].

Our results further confirm the hypothesis that therapists who started to use remote psychotherapy had better experiences with remote psychotherapy than previously expected; this was more pronounced for web-based psychotherapy than for telephone-based psychotherapy. This result is supported by previous studies showing positive experiences of therapists using remote psychotherapy [43-45]. As reviewed by Connolly et al [2], the functionality and ease of use of psychotherapy provided via videoconferencing was reported to be a “pleasant surprise.” The generally positive experiences with remote psychotherapy can be explained by benefits reported previously by therapists, such as improved access to therapy and less traveling time [41]. However, the special situation of the COVID-19 pandemic may have further benefitted the favorable rating of remote psychotherapy, as it represented the only way to maintain psychotherapy while adhering to the protective measures against COVID-19. In general, psychotherapists are the individuals who determine the frequency of use of remote psychotherapy. The finding that psychotherapists experience remote psychotherapy more positively than previously expected suggests that it is important to provide a practical experience (already in training); this finding is also in line with a previous study on changing attitudes of health care providers after first-time use of telemedicine [12].

The findings were also influenced by the participants’ therapeutic orientation. In contrast to previous findings, behavioral therapists reported less positive actual experiences compared to previous expectations and lower comparability of telephone-based psychotherapy to in-person therapy than psychodynamic therapists [28-30]. This result contrasts with our hypothesis, which assumed that psychodynamic psychotherapists would rate telephone-based psychotherapy more negatively than behavioral psychotherapists. Previously, it was assumed that psychodynamic therapists would particularly feel that they were losing valuable information through remote psychotherapy, even when using videoconferencing. The analysis of nonverbal behavior is of paramount importance in psychodynamic theory. Thus, information regarding behavioral or physiological changes (such as crossing or swinging a foot, muscle tension, or perspiration) is difficult to perceive in remote settings, even when using a camera [29]. However, our results do not support the assumption that this loss of information will have a detrimental effect on the attitudes and experiences of psychodynamic therapists toward remote psychotherapy. In contrast, psychotherapy via telephone, which causes a much stronger loss of information related to nonverbal behavior than videoconferencing, was rated more positively (regarding comparability to face-to-face therapy and experiences vs expectations) by psychodynamic therapists than by therapists of other therapeutic orientations. However, ratings of comparability to face-to-face psychotherapy were also higher for web-based therapy than for telephone-based therapy for psychodynamic therapists. On the other hand, the lowest ratings for telephone-based therapy (regarding comparability to face-to-face therapy and experiences vs expectations) were found for behavioral therapists compared to therapists of other therapeutic orientations. Because behavioral therapy is more focused on changing maladaptive behaviors [29], we assumed that those therapists would rate remote psychotherapy more positively than other therapists, as these maladaptive behaviors could be treated similarly in remote and face-to-face therapy. However, it is possible that the observed differences among therapeutic orientations are confounded by other factors, such as differences in gender, age, or therapeutic experiences, as those factors have been speculated to be potential moderators of attitudes toward remote psychotherapy [2,46]. To rule out any confounding effect of the observed differences concerning age and experience (ie, the younger age and shorter period in the profession of behavioral therapists), those variables were included as covariates in ANCOVAs. However, even when conducting ANCOVAs, differences among therapeutic orientations remained significant. Thus, further studies are required to investigate the potential reasons behind the observed differences.

The ratings of the comparability of web-based and in-person psychotherapy as well as the discrepancies in actual experiences and previous expectations regarding web-based psychotherapy did not differ regarding therapeutic background. This result rejects our hypothesis that behavioral psychotherapists would rate remote psychotherapy via internet more positively than psychodynamic therapists.

In general, web-based psychotherapy was rated more positively (regarding comparability to face-to-face therapy and experiences vs expectations) than psychotherapy via telephone; however, this was not the case for psychodynamic therapists, who reported their previous expectations to be equal to their actual experiences for both telephone- and web-based psychotherapy. This confirms our hypothesis and is likely due to the potential of videoconferencing to provide several pieces of information regarding nonverbal behavior and of its greater comparability to face-to-face settings than telephone-based psychotherapy, which results in a greater loss of information.

### Conclusions

The experiences of psychotherapists with remote psychotherapy were better than their expectations but not totally comparable to face-to-face psychotherapy with personal contact. Adequate mental health care is of high importance during the COVID-19 pandemic, and the provision of psychotherapy at a safe distance seems to be the obvious solution to ensure sufficient psychotherapeutic support. This study provides important insights regarding psychotherapists’ experiences with and expectations of remote psychotherapy during the COVID-19 pandemic. Future studies should also consider patient perspectives.

## Abbreviations

ANCOVA: analysis of covariance
ANOVA: analysis of variance
REDCap: Research Electronic Data Capture
RQ: research question
